# A Selective-Response Hypersensitive Bio-Inspired Strain Sensor Enabled by Hysteresis Effect and Parallel Through-Slits Structures

**DOI:** 10.1007/s40820-023-01250-y

**Published:** 2023-11-20

**Authors:** Qun Wang, Zhongwen Yao, Changchao Zhang, Honglie Song, Hanliang Ding, Bo Li, Shichao Niu, Xinguan Huang, Chuanhai Chen, Zhiwu Han, Luquan Ren

**Affiliations:** 1 Key Laboratory of Bionic Engineering (Ministry of Education), Jilin University, Changchun, Jilin 130022 People’s Republic of China; 2 Liaoning Academy of Materials, Liaoning, Shenyang 110167 People’s Republic of China; 3 Key Laboratory of CNC Equipment Reliability (Ministry of Education), Jilin University, Changchun, Jilin 130022 People’s Republic of China

**Keywords:** Bio-inspired strain sensors, Hysteresis effect, Hypersensitivity, Selective frequency response, Health monitoring applications

## Abstract

Flexible strain sensors are promising in sensing minuscule mechanical signals, and thereby widely used in various advanced fields. However, the effective integration of hypersensitivity and highly selective response into one flexible strain sensor remains a huge challenge. Herein, inspired by the hysteresis strategy of the scorpion slit receptor, a bio-inspired flexible strain sensor (BFSS) with parallel through-slit arrays is designed and fabricated. Specifically, BFSS consists of conductive monolayer graphene and viscoelastic styrene–isoprene–styrene block copolymer. Under the synergistic effect of the bio-inspired slit structures and flexible viscoelastic materials, BFSS can achieve both hypersensitivity and highly selective frequency response. Remarkably, the BFSS exhibits a high gage factor of 657.36, and a precise identification of vibration frequencies at a resolution of 0.2 Hz through undergoing different morphological changes to high-frequency vibration and low-frequency vibration. Moreover, the BFSS possesses a wide frequency detection range (103 Hz) and stable durability (1000 cycles). It can sense and recognize vibration signals with different characteristics, including the frequency, amplitude, and waveform. This work, which turns the hysteresis effect into a "treasure," can provide new design ideas for sensors for potential applications including human–computer interaction and health monitoring of mechanical equipment.

**Supplementary Information:**

The online version contains supplementary material available at 10.1007/s40820-023-01250-y.

## Highlights

- A bio-inspired flexible strain sensor with hypersensitivity and highly selective frequency response is prepared by styrene–isoprene–styrene combined with monolayer graphene.
- Benefiting from the structural design inspired by nature and hysteresis of viscoelastic materials, bio-inspired structures, and original materials' properties complement each other.
- The frequency recognition resolution of bio-inspired flexible strain sensor reaches 0.2 Hz, making it ideal for human–computer interaction and mechanical equipment health inspection.

**Supplementary Information:**

The online version contains supplementary material available at 10.1007/s40820-023-01250-y.

## Graphical abstract

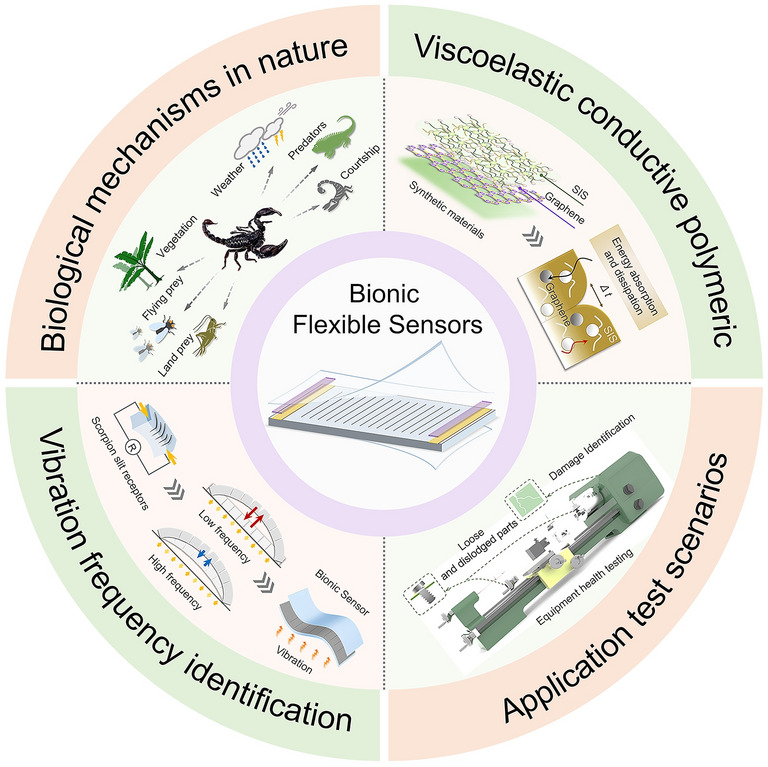

## Introduction

In recent years, flexible electronics have made significant progress. There is a growing range of applications, such as human health and motion monitoring [1–3], human–computer interaction [4–8], stretchable smart devices [9–11], and soft robotics [12, 13]. According to the signal conversion mode, pressure sensors can be classified as capacitive [14–16], piezoelectric [17, 18], frictional [19, 20], and piezoresistive [21–25] sensors. In particular, piezoresistive flexible sensors, which convert mechanical pressure signals into resistance changes, have received much attention relying on their advantages such as simple structure and fast response. The sensitivity of flexible sensors, as an important performance evaluation index, can be achieved by continuously improving the sensing structure or changing the functional materials [26–29]. However, the increased sensitivity is usually accompanied by high hysteresis [30], which is caused by internal and interfacial energy dissipation of the flexible material. Especially for piezoresistive flexible sensors applied in the field of frequency identification [31, 32], external vibration factors can cause deformation of the sensor, which in turn drives the internal and external friction of the polymer matrix or composite material. Hence, its frequency identification performance is strongly affected by the hysteresis of the material. That is to say, sensitivity and hysteresis are two coordinated parameters of flexible sensors. Therefore, it is challenging for flexible strain sensors to simultaneously achieve hypersensitivity and highly selective frequency response.

Researchers have made great efforts to balance the high sensitivity and selective frequency response of flexible sensors by changing the materials or developing suitable structures [33, 34]. Creating specific microstructures for the flexible sensor is an effective method to reduce the friction and adhesion on its surface, thereby can reduce the hysteresis while not affecting sensitivity. For example, a piezoresistive pressure sensor with uniform porous structures [35] was prepared to achieve a low hysteresis of 2%. A bionic flexible sensor with the flea’s structures [36] reduced the hysteresis to 1.3%. The hysteresis of a microfluidic flexible strain sensor combining liquid metal with a waveform channel structure [37] decreased to 1.02%. However, the imposition of additional specific structures results in poor repeatability. In addition, improving the molecular structures of flexible materials applied to sensors is another effective method to address the issues caused by hazardous hysteresis. For example, a method was developed to reduce friction and entanglement between molecules in viscoelasticity, benefiting from the bridging and swelling effects between different materials [38]. Introducing dynamic bonds between conducting polymers, such as hydrogen bonds [39, 40], can also help improve the mechanical robustness of flexible composite materials and reduce hysteresis. This method improves weak interactions by increasing strong covalent bonds between the conductive material and flexible polymer, but the synthesis process is cumbersome and difficult to repeat. In a word, current research has made significant progress in reducing the adverse effects of hysteresis. Unfortunately, it is still difficult to achieve both high sensitivity and completely avoid the low signal recognition resolution problem caused by the hysteresis of the flexible material itself. The coordinated effects of sensitivity and hysteresis have limited the potential applications and scope of flexible sensors.

After billions of years of evolution, creatures in nature possess almost perfect structures and surface functions. Nature has experimented with various solutions to its challenges and has improved the successful ones, which can provide ready answers to scientific and technical problems and inspire us with a series of novel designs and high-performance structures. For scorpions, their living environment contains a variety of vibrational information. In this respect, frequency is a key element for scorpions to discriminate external vibrational information. When the vibrational signals are transmitted to the slit receptors, a series of mechanical transduction leads to compressive deformation of the slit receptors. Facilitated by this function, scorpions can achieve precise identification of target signals among complex vibrational signals. This ingenious biological strategy can provide inspiration for the design of bionic sensors [41]. Recently, research on sensors with scorpion-inspired structures has been increasing [42]. At this point, the incorporation of bionic structures provides the sensors with praised excellent performance.

In this work, inspired by the scorpion slit sensor, a bio-inspired flexible strain sensor (BFSS) is prepared by constructing a through-slit array in the self-synthesized flexible conductive polymer. Specifically, the conductive polymer is made of monolayer graphene and styrene–isoprene–styrene block copolymer (SIS), which exhibits superior electrical and mechanical properties. It was verified that the conductive polymer has excellent stretchability (1600%) and good electrical conductivity (33.1 S m^−1^). In addition, the relationship between the hysteresis characteristics and the vibration frequency was characterized. At the same time, the vibration recognition function of BFSS was demonstrated. Tests yielded a frequency recognition resolution (0.2 Hz) and a frequency response range (<103 Hz) of BFSS, with the GF 30 times higher than the unstructured samples. Benefiting from the bio-inspired structures and viscoelastic materials, BFSS alleviates the contradiction between sensitivity and hysteresis, and possesses a high-precision frequency recognition function. The excellent performance of the BFSS allows it to distinguish between different tones of sound, judge the speed of mechanical equipment in operation and react to loose parts of the equipment. This study paves the way for designing next-generation flexible strain sensors for potential applications of human–computer interaction, structural health monitoring of precision equipment, and engineering failure detection.

## Experimental Section

### Materials

The scorpions were purchased from Hainan Province, China. The rearing environment was a constant temperature of 25–30 °C in a suitable incubator. SIS were purchased from Asahi Kasei Corporation. The SIS model is SIS-1106. Specifically, the SI block ratio is 15/85 and the SI content is 17%. Polyurethane (F0401) was purchased from Shenzhen Ji Ltd. Graphene was purchased from Suiheng Technology Co., Guangdong, China. Conductive silver pastes were obtained from Tiangong Accessories, Shenzhen, and Dow Corning Corp. Absolute ethanol (99.5%) was purchased from Beijing Chemical Works, China.

### Preparation of BFSS

Firstly, 1.6 g of graphene and 32 g of toluene (C_7_H_8_) solution were homogeneously mixed and subjected to magnetic stirring and ultrasonic vibration for 15 min, respectively. Next, 6.4 g of flexible material (SIS) was continued to be added to the mixed solution, with a 30-min ultrasonic vibration treatment. Then the mixed solution was placed on a magnetic mixer to receive 24 h of magnetic stirring, to make the solution well mixed. After that, the mixed solution is poured into a Petri dish and left to stand for 20 min, after which it is moved to a heating table to receive a constant heating at 25 °C for 24 h, to evaporate toluene (C_7_H_8_). After the treatment, the flexible conductive polymer was cured and molded, using a laser marking machine to cut into strips of conductive material. Next, parallel through-slit structures were prepared on top to mimic the parellel slit receptors of the scorpion. A through-slit allows for a larger bonding area on both sides of the slit structure and contributes more to the hysteresis effect than a non-through slit. At the same time, the design of through-slit can better comply with the tensioning at different frequencies. The number of strips of through-slit is controlled in the range of 7–15, with the base strip selected to meet the requirement of always having a central slit. At this time, the main part of the sensor was completed. In the next step, the sensor was connected to the wires. Conductive silver paste was applied to both ends of the main part of the sensor, followed by bonding of flexible conductive adhesive tape. Later, the sensors were put into an oven at a temperature of 80 °C for 30 min. After the drying was finished, a polyurethane film was applied to the surface of the sensor as a protective layer for the sensor. In this case, the F0401 waterborne polyurethane was uniformly applied to the outer surface of the sensor using sterile cotton. After that, it was placed indoors to dry naturally. Finally, a selective-response hypersensitive bio-inspired strain sensor was successfully prepared.

### Preparation of the Biological Specimens

Scorpions with normal physiological functions were selected. The surface was cleaned with ethanol and medical cotton balls. At this point, pay attention to the leg crevices and hairs. The patellas of the scorpions were dissected after the scorpions were anesthetized with ether. The patellas of the scorpion legs were dehydrated, after which they were fixed into the resin. The specimens were processed and cut along the direction perpendicular to the slit. Specimens were fixed on slides and sectioned specimens were studied using SEM (JSM-6700F, JEOL) and EDS (X-MaxN 150, Oxford, Britain). The morphology of the slit receptors was observed using a Super Depth of Field 3D microscope. All the animal treatments comply with the Chinese Law on the Protection of Animals. Ethical approval was given by the Animal Experimental Ethical Inspection, Jilin University.

### Measurement Characterizations

The surface topography analysis of SIS polymers is achieved through the operation of the Super Depth of Field 3D microscope (VHX-6000). SEM (JSM-6700F, JEOL) is employed for the analysis of the elemental composition within polymers and the morphological analysis of scorpion sensory organs. The tensile properties of all samples in this study were tested using a tensile testing machine (XLD-20E) at room temperature with a loading rate of 10 mm min^–1^. The bending fatigue tests were conducted using a custom-made testing system, and the sensor was bent or response time using positioning-controlled motorized linear stages (SURUGA SEIKI) for a basic performance test. A digital multimeter (34465A 61/2 digit, Agilent) was utilized to measure the resistance signal changes in the sensor to evaluate its sensing parameters such as sensitivity and frequency Identification reliability.

## Results and Discussion

### Design and Fabrication of Sensors

**Fig. 1:**
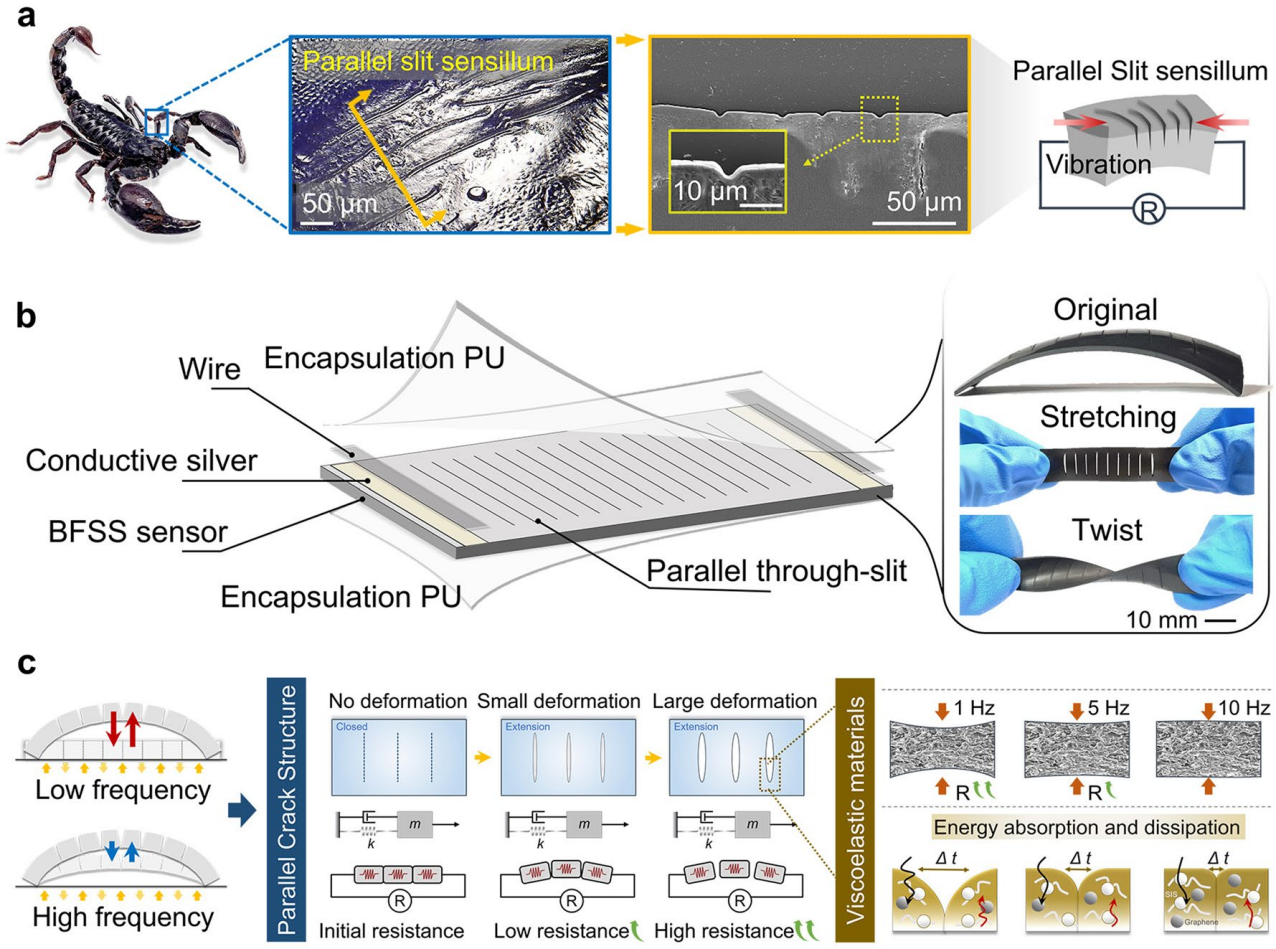
Design and fabrication of a bionic flexible strain sensor inspired by the scorpion’s slit sensillum. **a** Enlarged optical image of the scorpion's slit sensillum; SEM images of parallel slit structures of scorpion leg sensillum; Schematic diagram of the parallel slit sensillum. **b** Illustration of the bionic flexible strain sensor based on BFSS; Digital images of the bionic sensor. **c** Based on the parallel through-slit structures and viscoelastic materials, BFSS demonstrates different external morphology and internal energy changes when subjected to low-frequency and high-frequency vibrations

The design and fabrication of a bionic piezoresistive strain sensor, which draws inspiration from scorpion slit receptors, is shown in Fig. 1. It had been reported that scorpions, as arthropods with special living environments and habits, had evolved over a long period of time and the sensitivity of other receptors had been increasing. Howener the visual system of scorpions is highly degraded. It has been found that the micro-vibration slit receptors in the legs of scorpions have high sensing ability, can detect gravel disturbances within 20 cm of a sandy surface with a high attenuation factor, as well as identify the source of tiny vibrations as far as 50 cm [43]. Based on the scorpions’ highly sensitive sensing ability, we selected scorpions as the biological prototype to investigate their sensing mechanism and develop the bio-inspired flexible strain sensors. The location and surface morphology of the patellar suture receptors of the scorpion are shown in Fig. 1a. It is observed that scorpions have a staggered, nearly parallel, distribution of linear slits at the patella on the superficial cuticle. When external vibrations occur, the slit receptors are squeezed to generate biological signals (Figs. S9 and S10, Video S3). At this point, the scorpion can make judgments about external vibration signals through this leg slit. At the same time, the slit serves as an energy collector for vibration signals [44]. Inspired by the scorpion receptor, a parallel through-slit structural design is introduced into the development of the sensor (Fig. S11 and Video S3). The bio-inspired structures turn the hysteresis effect into an advantage. Ultimately, under the synergistic effect of the bionic parallel slit structures and the hysteresis effect of flexible materials, the bio-inspired flexible sensor possesses high-precision recognition of vibration frequency.

In Fig. 1b, a bionic sensor shape design inspired by the parallel slit sensing structure of a scorpion is shown, demonstrating the morphology of the prototype sensor under natural placement, stretching, and twisting (Video S1). The conductive part of the sensor is composed of a conductive material (Monolayer graphene) and a flexible material styrene–isoprene–styrene block copolymer (SIS) in a 1:4 ratio, and the detailed chemical molecular composition pattern is shown in Fig. S1 of the support material. SIS has a stable hysteresis, and its properties are not affected by mixing with graphene through solvents. To introduce the fabrication process of the sensor, as shown in Fig. S2. After the conductive polymer part is fabricated, the sensor is conneced to the wire. Next, conductive silver paste is applied to both ends of the strip sensor and flexible conductive adhesive tape is bonded. After waiting for fixation, a polyurethane film was applied to the sensor surface for surface protection of the sensor. Finally, we fabricated a bionic flexible piezoresistive strain sensor element with a thickness between 0.8 and 0.9 mm, a length of 30 mm a width of 10 mm, and a slit of 6 mm.

### Mechanism Analysis of Sensors

At this time, we design the bionic sensor, which is compliant and takes advantage of the inevitable hysteresis effect of flexible sensors, to achieve the function of accurate recognition of vibration signal frequency, with the assistance of the bionic parallel slit structure. The sensing mechanism of the sensor is demonstrated in detail in Fig. 1c. A model of the bionic sensor element with seven parallel through-slits is designed. An increase in the number of slits can further improve the sensitivity of the sensor. However, in this work, the number of slits is set to 7 to avoid the situation where the internal stress in the sensing material decreases after the number of slits increases, thereby affecting the hysteresis performance. When the sensor is stationary, the parallel through-slits are tightly closed under the preload of the polyurethane (PU) encapsulation layer. In the ideal state without the hysteresis effect, the slit structure of the sensor can be restored to a completely closed state after one vibration. Performing the next vibration stimulus, the initial resistance value (*R*_0_) of the sensor remains constant for each vibration. In the actual state, the hysteresis of the flexible sensor determines that it does not have instantaneous recovery after a strain stimulus, and the required recovery time increases with the increase in hysteresis. When stimulated by low-frequency vibrations, the sensor with a high hysteresis effect can achieve a greater degree of recovery, due to the longtime interval (*Δt)* between the previous vibration and the next vibration, during the time interval between two adjacent vibrations. The sensor also shows a relatively high change in resistance (*ΔR*) before and after the application of one vibration. When stimulated by high-frequency vibrations, the time interval (*Δt*) between two neighboring vibrations is very short. At this point, the slit structure of the sensor cannot close completely after opening, due to the high hysteresis effect of the flexible material. As a result, the initial resistance value (*R*_0_) increases during successive vibrations, and the corresponding value of the change in resistance (*ΔR*) before and after the sensor decreases during the course of one vibration. In the face of different vibration stimuli, the sensor exhibits different morphological changes corresponding to different resistance values. Here, when we design a flexible sensor with a large deformation, the parallel gap is opened to a large extent and therefore has a larger resistance value. Conversely, it has a smaller resistance value when a small deformation occurs. From the perspective of viscoelastic materials, low-frequency vibrations usually involve lower energy transfer rates, allowing more time for the material to absorb and dissipate energy and therefore have greater deformability. Similarly, high-frequency vibration stimulates a short time for energy conversion of the material, which leads to a lower deformation capacity of the material.

This bionic parallel through-slit design allows the sensor to rely on its hysteresis effect, which is difficult to eliminate. It has the feature of large slit tension under low-frequency vibration stimulation and small slit tension under high-frequency vibration stimulation, in the inherent frequency range. In addition, since the hysteresis phenomenon of the sensor is not suppressed, the sensitivity of the sensor remains relatively high benefiting from the bionic structures. When stimulated by small amplitude weak vibrations, it can exhibit stable and sensitive vibration responses. Based on the above, the sensor we studied has good vibration sensing and frequency recognition functions.

### Sensing Performance

**Fig. 2:**
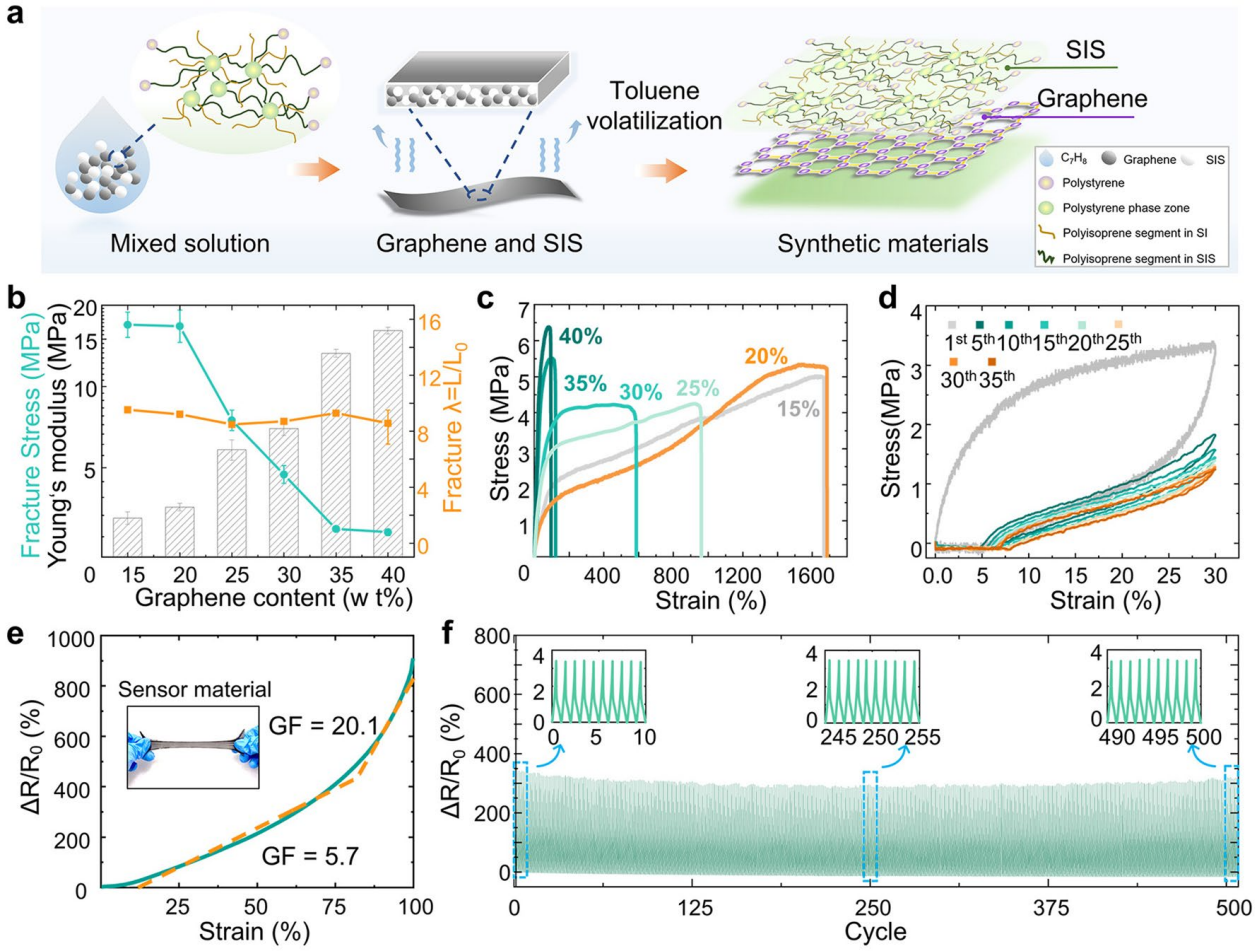
Characteristics of the performance of conductive polymers. **a** Preparation of BFSS sensor and schematic diagram of molecular structure of graphene and SIS. **b** Elastic modulus testing of conducting polymers with graphene content of 20%, 25%, 30%, 35% and 40%. **c** Tensile fracture testing of conductive polymers with graphene content of 15%–40%. **d** Tensile cycling test of a conductive polymer with 20% graphene content. **e** Sensitivity of conducting polymers with 20% graphene content. **f** Curve of the change in resistance of a conducting polymer with 20% graphene content at 500 cycles with 50% strain applied. The inset shows a close-up of the selected area

The sensing properties of the sensor material are illustrated in Fig. 2, including the elastic modulus, tensile rate, sensitivity and stability of the material. Among them, Fig. 2a shows the process of the sensor's material from mixed solution to the solid-state device, including material structure analysis. The structures of the flexible materials, including the monolayer two-dimensional honeycomb lattice structure of monolayer graphene, and the styrene–isoprene block structure of SIS. Elemental analysis of the material revealed that the material elemental composition was dominated by carbon and oxygen with a uniform distribution (Fig. S3). By using the non-polar solvent toluene to dissolve the graphene and SIS, these two materials can be well and evenly mixed (Fig. S4, Video S4). Figure 2b shows that the elastic modulus was tested for conducting polymers with 20%, 25%, 30%, 35%, and 40% graphene content, respectively. It can be seen from the figure that the difference in elastic modulus between 20% content (0.48 MPa) and 25% content (4.14 MPa) is about 3.67 MPa, and the difference in elastic modulus between 25% and 30% content (5.34 MPa) is about 1.20 MPa. This indicates that the 20% content of the conductive polymer has a prominent reduction, concerning the 25% content, in the modulus of elasticity. The modulus of elasticity is a measure of the degree of difficulty in producing elastic deformation, of a material. The experimental data suggest that the conducting polymer at this ratio has less difficulty in undergoing elastic deformation and is more viscoelastic. Similarly, hysteresis is more pronounced. Tensile fracture tests were carried out on conductive polymers with graphene contents of 15%–40% (Fig. 2c), aiming to obtain the maximum tensile rate of the composites at the corresponding content. By using a tensile testing machine, the test elements were stretched at a uniform speed of 10 mm min^-1^. The rectangular strips with an average length of 18.5 mm and an average cross-sectional area of 2 mm^2^. Five repeatability tests were performed for each ratio. It can be seen from the figure, in this range, the greater the percentage of elastic material, the better the tensile properties of the polymer, with a Gr: SIS mass ratio of 15% exhibiting the maximum stretching (1687%). However, its tensile curve is not very stable, and it does not differ much from the maximum stretching of the polymer, with a Gr: SIS mass ratio of 20% content (1667%, Video S2). Taking all factors into account, a polymer with a Gr: SIS mass ratio of 20% is the most reasonable for manufacturing sensors. Moreover, it ensures better tensile performance (Fig. S5). For the materials used to make the sensors, mechanical stability is important, we conducted cyclic loading tests with 300% strain on a conductive polymer, using graphene with 20% content. As shown in Fig. 2d, the test element was a rectangular strip of 18.5 mm in length and 2 mm^2^ in average cross-sectional area, which was stretched at a speed of 10 mm min^−1^. The first cycle was carried out for stress relief, and the stress–strain curves of the following 5~35 cycles stabilized, which proved its good elasticity and fatigue resistance.

Following this, we performed sensitivity tests on a conducting polymer with 20% Gr content, without applied structure, at 100% strain. Generally, sensitivity is considered one of the key parameters of strain sensors, reflecting the relationship between the relative resistance change and the applied strain. The slope reflects the representative parameter used to evaluate the sensitivity, which is defined by the metrology factor (GF). As shown in Fig. 2e, *R*_0_ is the initial resistance of the conductive polymers,* ΔR* represents the change between the real-time resistance and the initial resistance, and *Δε* is the applied strain. The GF value of the sensitivity of the flexible sensor can be calculated by equation: ${\text{GF }} = { }\left( {\Delta R/R_{0} } \right)/\Delta \varepsilon$. As the data show, the non-applied structure exhibits a sensitivity of about 20.1 (*Δε*: 0.3%–1%). To verify the stability of the conductive polymer, a 20% Gr content conductive polymer was tested for real-time resistance response in tension at a fixed strain. A certain size of the conductive polymer was taken, coated with a conductive silver paste layer on both sides and glued with conductive tape to make the sensor. Finally, it was loaded and unloaded 500 times repeatedly at 50% strain using a stepper motor. The results show that the real-time *ΔR/R*_*0*_ exhibits cyclic variations, with the conductive polymer still maintaining excellent performance, verifying the durability and stability of the conductive polymer at 20% ratios (Fig. 2f). In addition, we also performed 1000 resistance cycling tests at 20% strain and other similar tests, the electrical performance remained good. This is important for practical applications (Fig. S7). In addition, we tested the conductivity, volt-ampere characteristics, and response time of conducting polymer (Fig. S6).

**Fig. 3:**
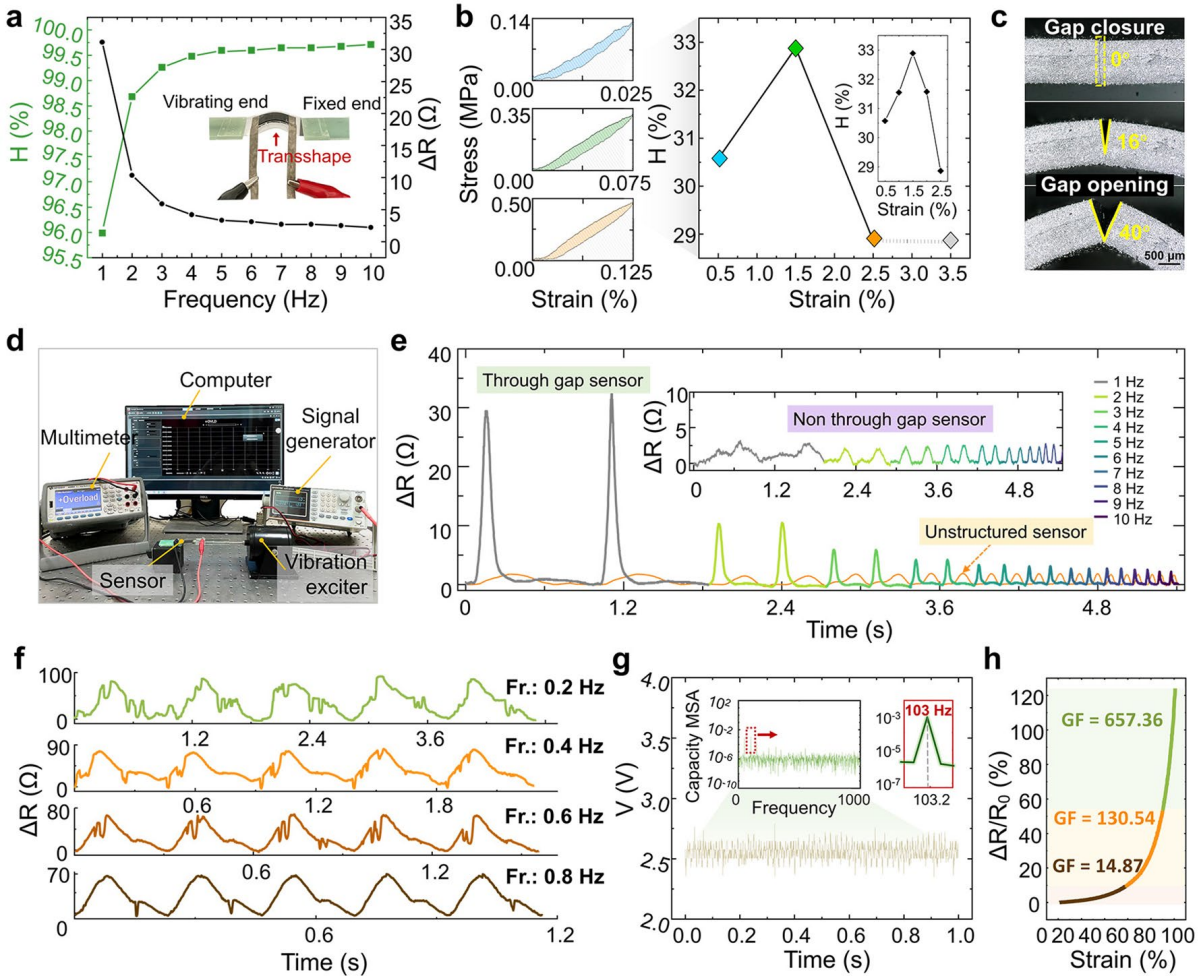
Hysteresis coefficient and frequency identification performance test. **a** Resistance change of the sensor at vibration frequencies from 1 to 10 Hz, with corresponding hysteresis coefficient (*H*%). **b** 0.5~2.5% tensile strain curve of the sensor; Hysteresis coefficient (*H*%) of the sensor in a tensile strain state of 0.5% -2.5%. **c** Enlarged optical image of the sensor with parallel through-slits. **d** Test environment for frequency identification performance testing. **e** Relative change in resistance of the sensor in the vibration range of 1–10 Hz, designed as a comparison with the test results of the sensor without parallel through-slit structure. The inset shows the test results of the sensor with a non-through-slit structure in the same environment. **f** Relative change in resistance of the sensor in the vibration frequency range of 0.2, 0.4, 0.6, and 0.8 Hz. **g** Frequency response test of the sensor. **h** Sensitivity testing of sensors

In the next step, we applied a bionic parallel gap structure to the conducting polymer and tested its performance in terms of frequency, as shown in Fig. 3. According to the available studies, the elastomeric materials applied to flexible sensors, such as PDMS, Ecoflex, and SEBS. When they are subjected to external forces, the deformation and recovery of the elastomer need to overcome certain internal molecular forces. This will take some time. Therefore, the strain of these elastomers will always lag behind the applied stress, with dynamic hysteresis occurring during loading and unloading. We analyzed the hysteresis performance of the sensor from two aspects. One is to calculate and plot the hysteresis analysis curve from the relationship, using an excitation table to emit a fixed frequency vibration, between the frequency to which the sensor is subjected and the resistance. The second is to analyze the relationship between stress–strain and hysteresis. The tensile state of the viscoelastic conducting polymer itself is tested using a tensile testing machine. The first step is to start from the function of frequency testing of the sensor itself, testing the degree of resistance change and calculating the hysteresis coefficient (*H*%), for different frequencies (1–10 Hz) with the same amplitude of 0.2 mm, this is shown in Fig. 3a. That is $H{\text{\% }} = (\Delta R_{{{\text{max}}}} - \Delta R_{F} )/\Delta R_{{{\text{max}}}} \times 100{\text{\% }}$. *ΔR*_max_ is the difference between the resistance of the sensor when the parallel slit structure is fully opened and left unstretched, compared to the resistance of the sensor when the slit is fully closed. *ΔR*_F_ is the change in resistance before and after the vibration response at different vibration frequencies. The data show that the hysteresis coefficient varies from 96.0 to 99.5% in the frequency range of 1–3 Hz. After the vibration frequency is higher than 3 Hz, the hysteresis coefficient tends to stabilize and becomes greater than 99.5%. Analyzing the curves, it can be seen that the hysteresis coefficient and resistance change are in opposite directions. As the applied vibration frequency increases, the hysteresis of the sensor increases. At the same time, the value of resistance change decreases. It is worth discussing that both show a simultaneous jump/sudden drop at the lower frequency stages and both stabilize at the higher frequency stages. This is consistent with our expected assumption, that the resistance change is sensitive at low frequencies and insensitive at high frequencies. Starting from the material of the sensor itself, the flexible conductive polymer is tested in tension at small strains. Using the feature that the stress–strain curves in the loaded–unloaded state do not overlap, to build a dynamic hysteresis curve, i.e., *H*% = *ΔS/S*_*J*_ × 100%. Where *ΔS* is the difference between the area of the loading curve (*S*_*J*_) and the area of the unloading curve (*S*_*X*_), *S*_*J*_ − *S*_*X*_ is the area of the hysteresis curve. Figure 3b shows that the hysteresis coefficient tends to increase in the strain range of 0.5–1.5%, reaching a maximum of about 33%, decreasing in the strain range of 1.5–2.5%, and stabilizing after greater than 2.5%. Refinement of the hysteresis coefficients in the range of 0.5–2.5% strain, the results show that the hysteresis coefficients corresponding to 1 and 2% strain are lower than those at 1.5% strain, in accordance with the overall curve direction. Analysis of the curves can be obtained that the hysteresis of the flexible conductive polymer at Gr: SIS=1:4 ratio does not increase with increasing strain. The growth of hysteresis coefficient in the initial stage of stretching is due to the energy loss caused by the destruction of graphene structure being faster than the energy consumed by SIS network stretching. When the initial graphene structure is completely destroyed, the hysteresis coefficient reaches a maximum value. Then the hysteresis coefficient decreases, because the change in energy loss within the material in the next stretching range is slower than the energy consumed by the stretching. So the overall data curve shows an increase and then a decrease. The above characteristics have positive significance for the sensor in this work. It provides a basis for the sensor to realize the frequency recognition function in a weak vibration environment.

The frequency recognition mechanism of the sensor is demonstrated in Fig. 3c. A super depth-of-field shot of the sensor's bionic slit structure is taken to observe the morphological characteristics when the slit opening angle is at 0°, 16°, and 40°. Comprehensive above sensor hysteresis performance and the relationship between the indicators, we test the frequency identification accuracy of the sensor. By applying vibration of different frequencies with fixed amplitude to the sensor through the excitation table, the vibration deformation state is simulated. While the resistance value of the sensor is monitored, the resistance change value of the sensor under the vibration stimulation of different frequencies. The operating state is shown in Fig. 3d. The test results show that the resistance change is about 30 Ω under 1 Hz vibration stimulation and decreases with increasing frequency. In 10 Hz case, the resistance change is about 2 Ω. The resistance change decreases slowly as the frequency increases. To verify the reliability of the data, we also tested the frequency recognition capability of the structured sensor without parallel gaps, finding that the structureless sensor could not make accurate recognition of frequency. We also designed a non-penetrating slit sensor and tested its frequency recognition capability. As shown in the inset figure, the non-penetrating slit sensor could not achieve accurate identification for frequencies under the same frequency range (Fig. 3e). The above test results are in correspondence with the increasing hysteresis coefficient of the sensor in the process of detecting the vibration frequency, and the increasing trend is gradually flattened. Next, we further tested the sensor frequency recognition function in a much low-frequency state. In Fig. 3f, vibration stimuli with same amplitude (0.2 mm) and different frequencies were applied to the sensor. The results show that the resistance change interval is about 0–100 Ω at low frequencies (0.2 Hz). As the frequency increases, the resistance change interval decreases. At a frequency of 0.8 Hz, the resistance interval decreased to 0–70 Ω. Therefore, it can be analyzed that the sensor in the low-frequency range (less than 1 Hz), can make accurate identification of the frequency interval of 0.2 Hz. For this reason, it is suitable for complex environments of small low-frequency vibration identification. In addition, we tested the response of the sensor for vibration waveform and vibration amplitude. As shown in Fig. S8, indicating that the sensor has good performance in vibration waveform recognition and vibration amplitude recognition. The frequency response performance of the sensor was tested by connecting a signal generator, power amplifier, and shaker and measuring the vibration frequency response range of the sensor. In Fig. 3g, the test data shows that the sensor can respond to vibration signals with frequencies up to 103 Hz. At the same time, we tested the sensitivity of the sensor at 100% strain, which possesses a parallel slit structure with 20% Gr content. The test data show (Fig. 3h) that the GF value (*ΔR/R*_0_)/*Δε*) is 14.87 in the 0–57% strain range, up to 130.54 in the 57–89% strain range, and up to 657.36 at 89–100% strain. The analysis showed that the GF value of the sensor increased by as much as 30 times compared to the conductive polymer without the parallel slit structure under the same strain conditions. Therefore, the introduction of the parallel slit structure has a positive effect on the sensitivity of the sensor.

### Application of Sensors

**Fig. 4:**
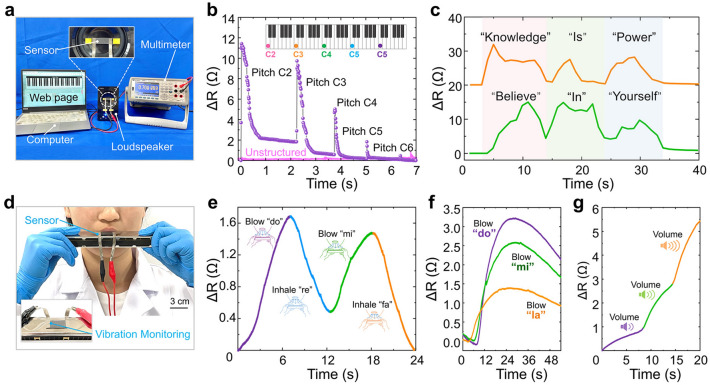
Micro-acoustic vibration testing of BFSS. **a** Test environment for sensing the vibration frequency. **b** Relative resistance changes of the sensor in response to different pitches of the same syllable. **c** Relative resistance changes of the sensor in response to different statements. **d** Photograph of the sensor performing the harmonica vibration test. **e** Relative resistance changes in the sensor in response to the four syllables "do," "re," "mi," and "fa." **f** Relative resistance changes of the sensor in response to the syllables "do," "mi" and "la." **g** Relative resistance changes in the sensor in response to different harmonica volumes

Given the sensor's excellent sensing performance and the physical characteristics of this design, the sensor can be well suited for various scenarios with small vibration signals from its frequency recognition perspective. The sensor was attached to the speaker diaphragm of a small stereo in a closed-fit manner, with the stereo connected to a laptop computer (Fig. 4a). Using the simulated piano software on the computer, click the buttons of different simulated piano keys at intervals in order from bass to treble. Since the vibration frequency of the sound body can determine the pitch, the higher the frequency, the higher the corresponding pitch; the lower the frequency, the lower the corresponding pitch. So the audio speaker from bass to treble sounding process, the speaker diaphragm showed the vibration frequency from high to low. Figure 4b shows the electrical signal data curve exhibited by the sensor when it received the same syllable (do) from the speaker diaphragm. At the same volume, with different pitches of the vibration signal. The degree of resistance change is used to measure the output signal of the sensor. As the vibration frequency increases, the value of the sensor's output signal (*ΔR*) tends to decrease. The lower the input vibration frequency, the greater the corresponding change in sensor resistance (*ΔR*). This is consistent with the working mechanism of the sensor designed in this work. It means that the higher the vibration frequency sensed by the sensor, the higher the hysteresis effect caused by the viscoelasticity of flexible materials. As a result, the smaller the open deformation of the sensor's parallel slit structure between the end of one vibration and the beginning of the next, the smaller the change in resistance during the vibration. Similarly, the unstructured sensor was installed and tested in the same application environment. The data curve was flat, indicating that the unstructured sensor didn’t possess tone recognition capability. Figure 4c shows the use of computer speech software to say the phrases "knowledge is power" and "believe in yourself", which showed that the sensor can effectively distinguish the pronunciation of different English words. In addition, the sensor was attached to the surface of the harmonica and the frequency recognition function of the sensor was tested by blowing the harmonica (Fig. 4d). Figure 4e shows the frequency recognition function of the sensor when the four syllables of "do", "re", "mi" and "fa" were played, different resistance change (*ΔR*) trends of the sensor after playing the four syllables. In the process of harmonica playing, the "do" and "mi" syllables need to blow, and the "re" and "fa" syllables require aspiration. The corresponding data curves showed a smooth upward trend during blowing and a smooth downward trend during inhaling. It shows that the sensor can distinguish between "inhalation vibration" and "exhalation vibration," which is a very interesting phenomenon. The analysis showed that, under the influence of the hysteresis performance of the sensor, the long vibration signal from the harmonica could be reflected in the data curve with a slow rise and fall. It was different from the sudden increase of the data curve corresponding to the short sound from the horn (Fig. 4b). The application of the sensor on the harmonica was tested as shown in Fig. 4f, where the three syllables "do", "mi" and "la" were played in turn. It was found that as the pitch of the syllable increased, the vibration frequency of the harmonica surface also gradually increased. At the same time, the corresponding output signal curve of the sensor showed a smaller and smaller value of the maximum resistance change (*ΔR*), while the slope of the curve also decreased and the rate of rise of the curve was getting lower. Figure 4g shows the same syllable of the harmonica played using different forces. The data showed that the louder the volume, the greater the amplitude of the vibration signal to which the sensor is exposed. In this regard, the output signal curve exhibits greater jumps. This indicates that the sensor can be very sensitive to the amplitude of the vibration signal. These signal trends can effectively prove, the sensor of this design has the sensitive ability to distinguish between low-frequency vibration and high-frequency vibration. Overall, the above data show that the presently designed sensor can effectively distinguish sounds with different vibration frequencies, as well as can be applied to the fields of vibration environment monitoring and speech recognition.

**Fig. 5:**
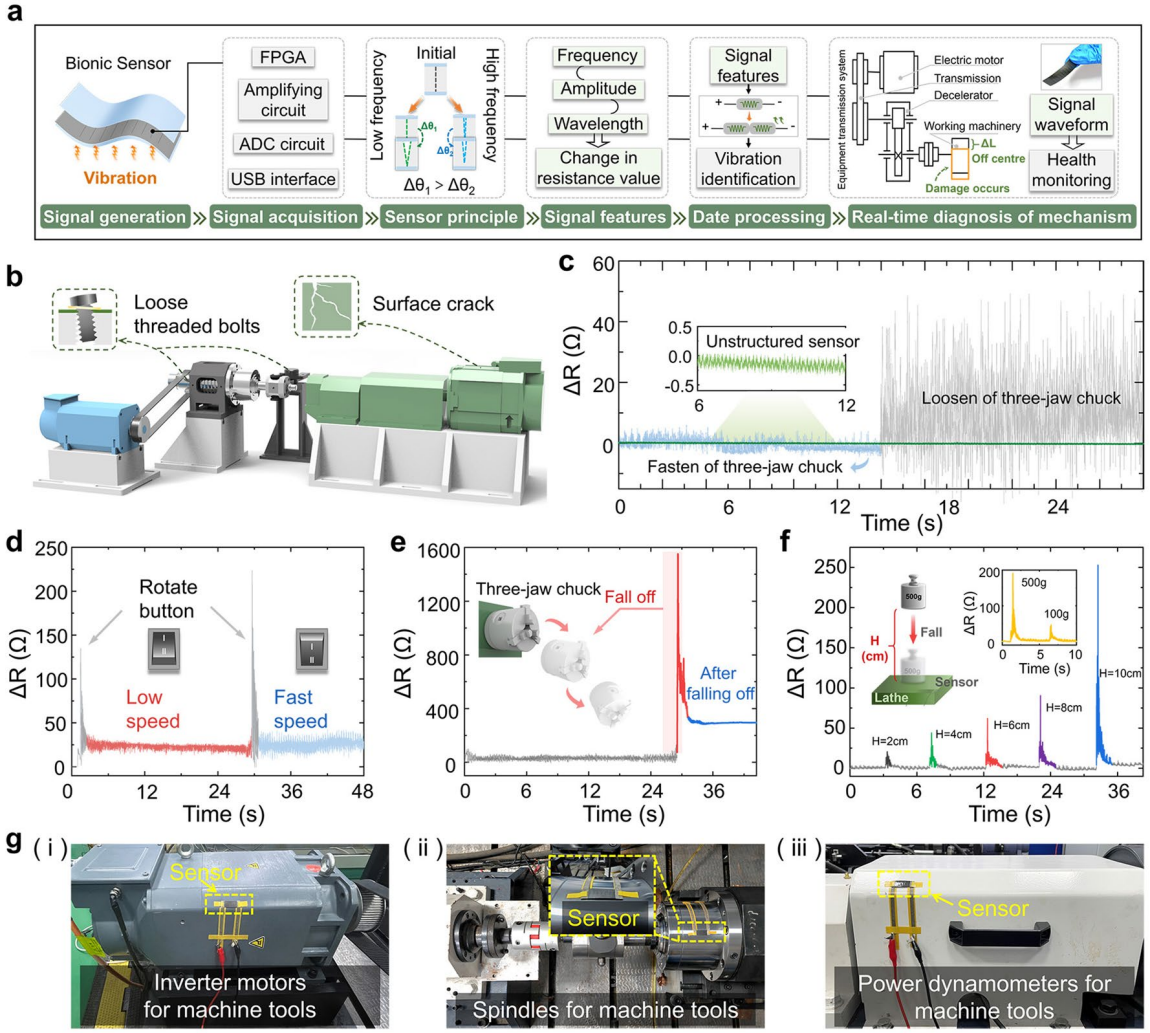
Application of the BFSS in the field of health inspection of machinery and equipment. **a** Vibration signal sensing system, including signal generation and acquisition, feature extraction, data processing, and real-time diagnosis. **b** Schematic diagram of the sensor used to detect the health condition of the machine tool. **c** Sensor installed on the machine to monitor the normal operation of the machine and the loosening of the bolt in real time, designed without parallel through-slit structure for comparison. **d** Sensitivity test of the sensor to the speed of the machine part movement. **e** Machine part shedding is monitored by the sensor. **f** Sudden disturbances at different impact speeds on the machine are tested by the sensor. **g** Photograph of the sensor monitoring machine vibration

The sensor designed here has good environmental adaptability to ensure effective work in complex noise environments, therefore it is suitable in areas such as health monitoring of mechanical equipment. In the process of health detection of mechanical equipment, a vibration signal sensing system was established with the specific steps in Fig. 5a. Including signal generation and acquisition, feature extraction, data processing, and real-time diagnosis. After the vibration signal is generated and transmitted to the surface of the sensor, high-speed signal acquisition is performed through the FPGA system. Data characteristics such as frequency, amplitude and wavelength in the vibration signal of the sensor are captured. Finally, it is converted into a resistance signal and output through data processing. During this period, if the machinery and equipment have a sub-healthy state, the vibration signal transmitted changes. This is followed by a simultaneous change in the output signal (*ΔR*) of the sensor, so that the health condition of the machinery and equipment can be judged. This health real-time detection function is suitable for machine tool part loosening detection, part shedding detection, surface crack detection, etc. (Fig. 5b). The vibration curve of the machine operation was monitored. In Fig. 5c, when the machine was in standby mode, the sensor could sense the small vibration brought by the motor operation in standby mode. At this point, a smooth output resistance change curve, with the resistance value change range was around 0.1 Ω. When the machine starts normal operation, the output resistance curve of the sensor compared with standby changed significantly. At this time, the resistance value changes in a range of about 5 Ω. The machine components are in normal working condition, showing no loosening and shedding phenomenon. Next, a state in which the bolts of the three-jaw chuck fixed on the machine are loosened is artificially created. According to the resistance curve of the sensor output, the sensor shows a large difference in the value of the resistance change. This is because the vibration state of the machine tool during operation after the three-jaw chuck is loosened is affected by the loosening of the bolts. As a result, the vibration condition presents a difference from that during normal operation. Usually manifested as the whole machine body vibration amplitude became larger, and intermittently appear with low-frequency large amplitude vibration signal characteristics. Here, the sensor relied on its sensitive electrical properties and excellent flexibility to provide real-time monitoring of the machine tool's operational health. In Fig. 5d, we again tested the sensitivity of the sensor to the running speed of the machine part. Here, we started from the machine standby state and turned the speed adjustment button, when the output curve exhibited a sudden peak due to the human disturbance factor. After the end of the artificial disturbance, the machine tool's three-jaw chucked in accordance with the low-speed gear operation. At this point, from the output curve could be seen in the resistance change value of about 9 Ω. Then turned the speed adjustment button to adjust the three-jaw chuck to high-speed gear operation. When the end of the artificial disturbance, it could be seen that the resistance change value of the output curve was around 22 Ω. It can be seen that the resistance change interval of low-speed operation had a big difference. As the above situation shows, the sensor designed in this case can identify the speed of running parts of mechanical equipment. In order to verify that the sensor has the function of sudden damage recognition of mechanical equipment, we artificially created the mechanical equipment damage environment. The purpose of this is to verify the damage recognition function of the sensor.

In Fig. 5e, all three fixing bolts of the three jaw chuck of the machine tool are loose while making the machine high-speed operation. After a period of time, the three-jaw chuck suddenly fell off under the action of centrifugal force. At this point, the output resistance of the sensor changed suddenly at the moment of disengagement. After this, it gradually stabilized. These data showed that the three-jaw chuck shedding made the machine produce instantaneous large vibration. The vibration signal at the sensor surface is detected in the form of resistance change value. Therefore, it can be judged that the presently designed sensor is useful for the identification of sudden damage to mechanical equipment. Finally, we verified the sensor's performance in detecting sudden disturbances with different impact velocities. We performed the tests with two different variables, as shown in Fig. 5f. By dropping a 500 g weight on the machine tool platform from different heights to achieve different speeds of vibration impact, the reference formula $v = \sqrt {2gh}$. It could be seen that as the impact speed increased, the output resistance changed the value of the sensor also showed an increase, so it could indicate that the sensor can judge the sudden disturbance speed. Similarly, by controlling the weight drop height and changing the weight mass, it is found that the sensor can still discriminate the impact situation of different weights. The sensor was installed in different parts of the machine tool to monitor the vibration of the machine tool, as shown in Fig. 5g. The above data show that the sensor has a sensitive detection function for sudden disturbances occurring on the machine tool, which can be applied to the field of machinery and equipment health inspection, as well as to make a judgment on the degree of damage or abnormal operation occurring on the equipment.

## Conclusions

In summary, inspired by the scorpion slit receptor, a bio-inspired flexible strain sensor was prepared. Specifically, BFSS is prepared by a self-synthesized conductive polymer consisting of monolayer graphene and SIS. The conductive polymer possesses superior electrical conductivity (33.1 S m^−1^) and stretchability (1600%). By combining the bio-inspired parallel slit configurations and flexible viscoelastic polymer materials, BFSS can effectively alleviate the contradiction between sensitivity and frequency recognition resolution. Accordingly, BFSS achieves both hypersensitivity and precise frequency recognition. The sensitivity tests at 100% strain were carried out to evaluate the sensitivity of BFSSs. It was found that the peak gage factor of the BFSSs is 657.36 under the same conditions, which is a 3170% increase compared to the flat flexible strain sensor (20.1). Besides, the different hysteresis performance of BFSSs for high-frequency and low-frequency vibrations is indirectly expressed as a different output signal (*ΔR*). We can effectively distinguish the vibration frequency with an interval of 0.2 Hz. According to the above results, the sensor is capable of accurately identifying minuscule vibrations in complex environments, which can be utilized in the field of weak vibration signal recognition such as piano pitch recognition and syllable detection. Furthermore, the high-precision frequency recognition function of BFSS enables it to be used as a health detecting device to monitor the status of various mechanical devices in real time. This study provides new inspiration and insight for improving the hypersensitivity and highly selective frequency response properties of flexible strain sensors for practical applications in human–computer interaction, sophisticated equipment health monitoring, and engineering failure detection.

## Supplementary Information

Below is the link to the electronic supplementary material.

Supplementary file1 (MP4 16501 KB)

Supplementary file2 (MP4 20797 KB)

Supplementary file3 (MP4 5650 KB)

Supplementary file4 (MP4 5032 KB)

Supplementary file5 (PDF 929 KB)
