# Supplementary material for: A Selective-Response Hypersensitive Bio-Inspired Strain Sensor Enabled by Hysteresis Effect and Parallel Through-Slits Structures
Source: Nanomicro Lett. 2023 Nov 20;16:26. doi: 10.1007/s40820-023-01250-y (PMC10661685; doi:10.1007/s40820-023-01250-y)
Supplement: Supplementary file 5 — Supplementary file5 (PDF 929 KB) [file 40820_2023_1250_MOESM5_ESM.pdf]

Supporting Information for

A Selective-Response Hypersensitive Bio-Inspired Strain Sensor Enabled by Hysteresis Effect and Parallel Through-Slits Structures

Qun Wang[1], Zhongwen Yao[1], Changchao Zhang[1], Honglie Song[1], Hanliang Ding[1], Bo Li[1,2,*], Shichao Niu[1,2,*], Xinguan Huang[3], Chuanhai Chen[3], Zhiwu Han[1,2,*], and Luquan Ren[1,2]

[1] Key Laboratory of Bionic Engineering (Ministry of Education), Jilin University, Changchun, Jilin, 130022, P. R. China

[2] Liaoning Academy of Materials, Liaoning, Shenyang, 110167, P. R. China

[3] Key Laboratory of CNC Equipment Reliability (Ministry of Education), Jilin University, Changchun, Jilin, 130022, P. R. China

* Corresponding authors. E-mail: boli@jlu.edu.cn (Bo Li); niushichao@jlu.edu.cn (Shichao Niu); zwhan@jlu.edu.cn (Zhiwu Han)

Supplementary Figures

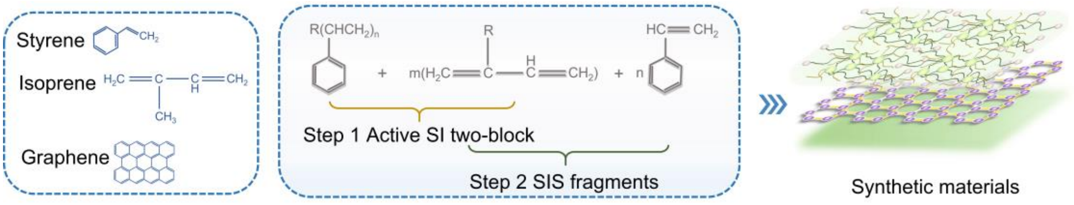

Fig. S1 Chemical molecular composition diagram of graphene and SIS

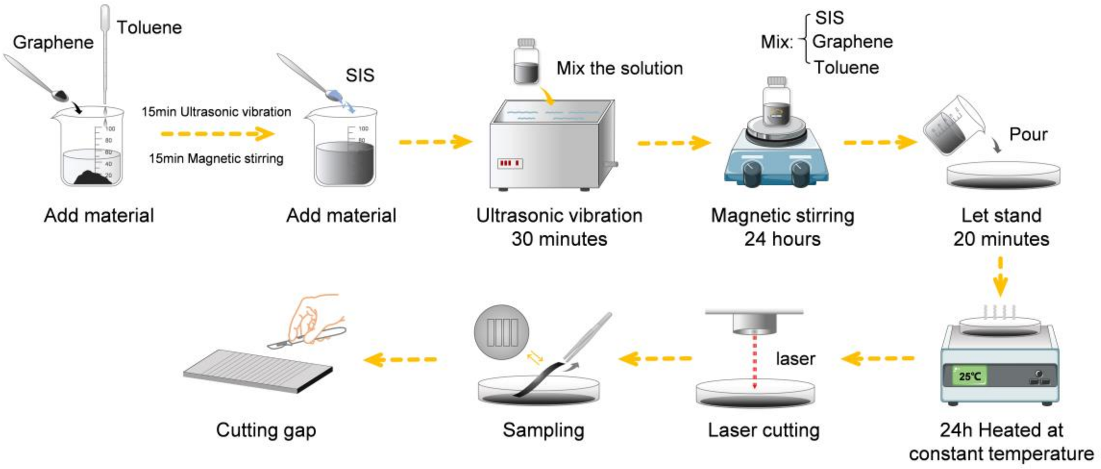

Fig. S2 Diagram of bionic flexible sensor fabrication process. This includes mixing of materials, stirring of suspensions, evaporation of liquids, and cutting structures

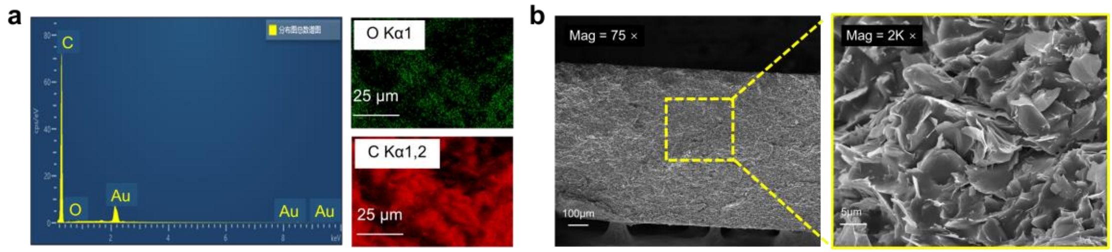

Fig. S3 Energy dispersive X-ray spectroscopy (EDS) plots highlighting the elemental distribution of the conductive polymers. (**a**) EDS of the material of the sensor. (**b**) SEM images of the sensor materials

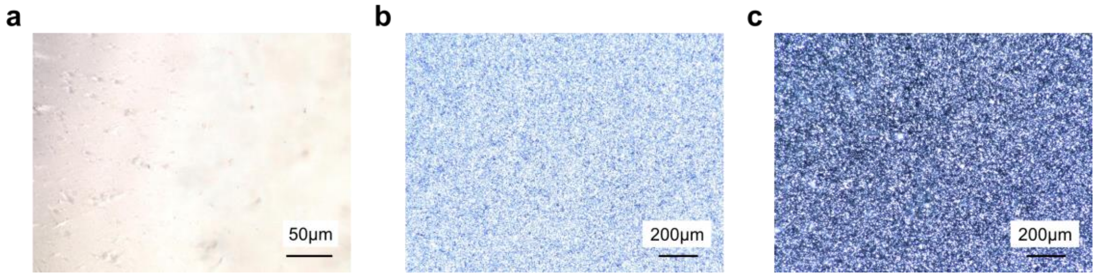

Fig. S4 Solution state in material preparation, photographed by Ultra-Depth Three-Dimensional Microscope. (**a**) Mixed state of SIS and toluene. (**b**) Mixture of graphene and toluene. (**c**) Mixture of SIS, graphene and toluene

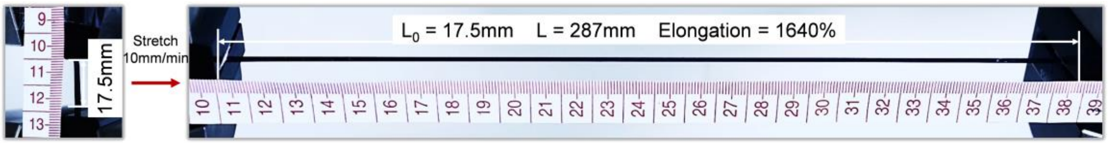

Fig. S5 Conductive polymers with 20% graphene content have great tensile properties, using a tensile tester to stretch the sample at 10 mm/min, showing 1640% tensile properties

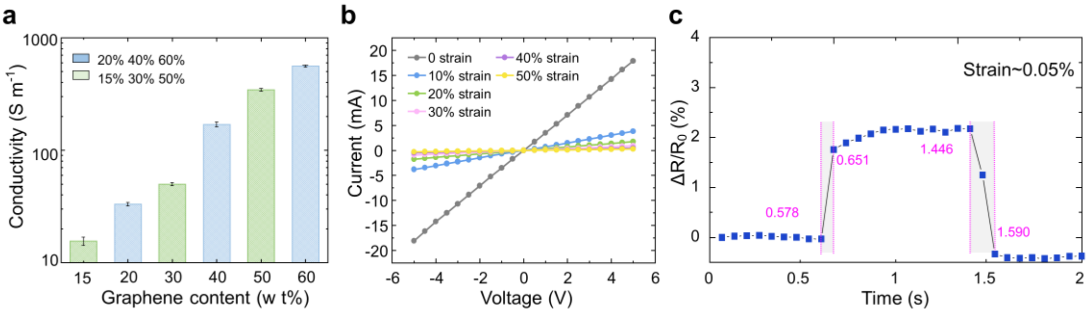

Fig. S6 Performance testing of conductive polymers. (**a**) Conductive polymers with graphene content of 15%, 20%, 30%, 40%, 50%, and 60% are tested for conductivity. (**b**) Volt ampere characteristic properties of conducting polymers with 20% graphene content are tested at no strain, 10%, 20%, 30%, 40%, and 50% strain. The volt ampere characteristic curves tend to be linear. (**c**) Time response of a conducting polymer with 20% graphene content over a strain range of 0-0.05%

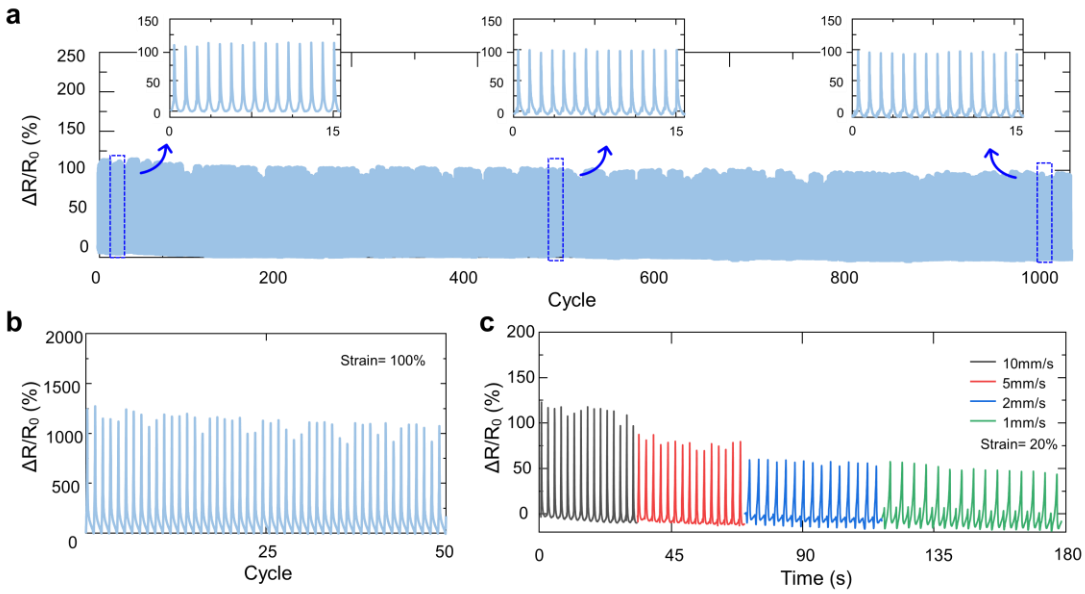

Fig. S7 Performance testing of conductive polymers. (**a**) Curve of resistance cycling of polymer with 20% graphene content undergoing 1000 cycles of 20% strain. (**b**) Curve of resistance cycling of polymer with 20% graphene content undergoing 50 cycles of 100% strain. (**c**) Curve of resistance cycling of polymer with 20% graphene content undergoing at 20% strain with different tensile speeds

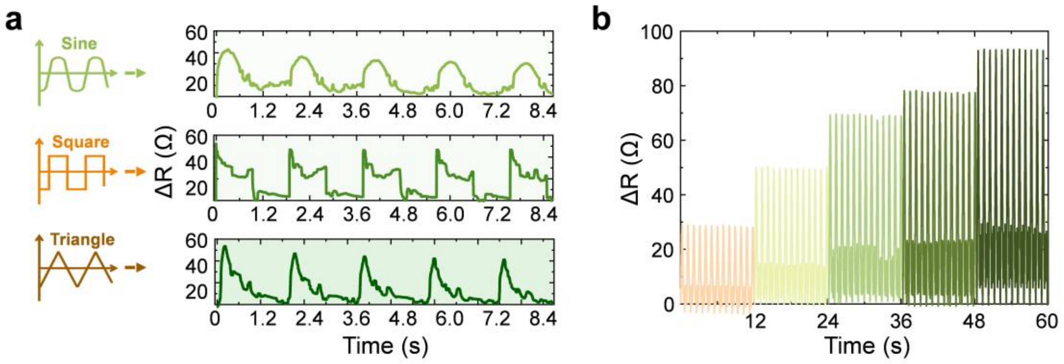

Fig. S8 Performance testing of the sensor. (**a**) Testing of the waveform recognition function of the sensor. (**b**) Testing the amplitude recognition function of the sensor

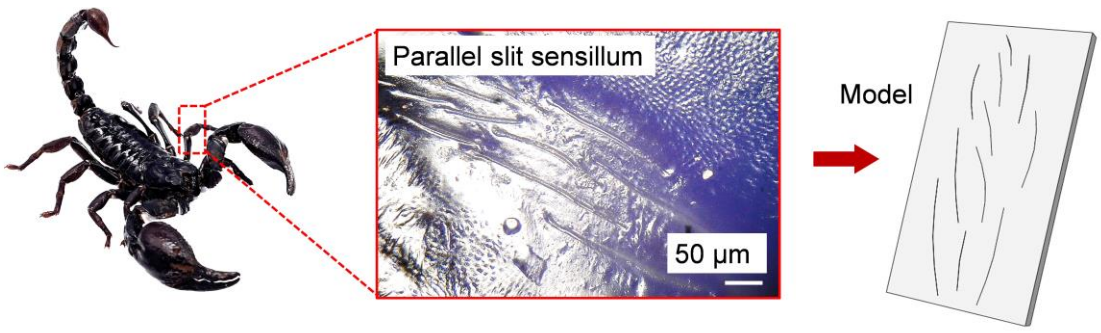

Fig. S9 Design ideas for the sensor structure

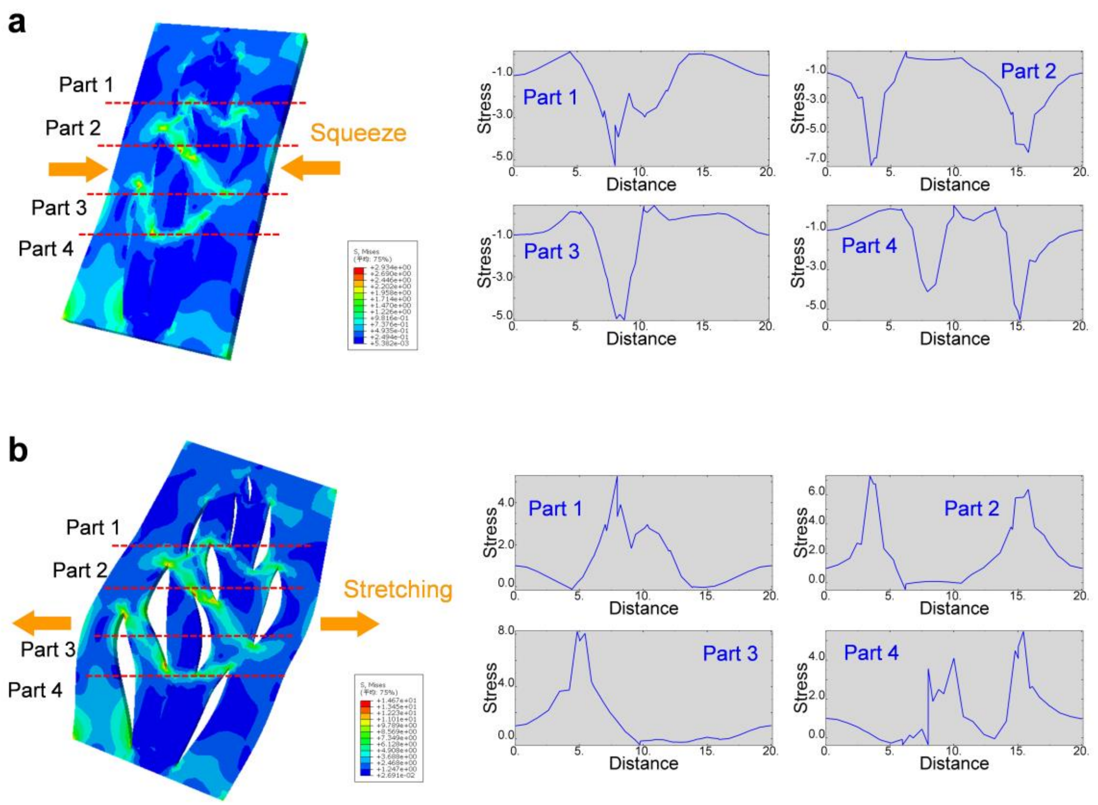

Fig. S10 Building a finite element model to mimic the slit sensillum of the scorpion. Simulating mechanical vibration stimuli in a real situation, surface tension and surface pressure are applied to the model, and stress analysis is performed on four regions. (**a**) Extrusion testing of the model. (**b**) Tensile testing of the model

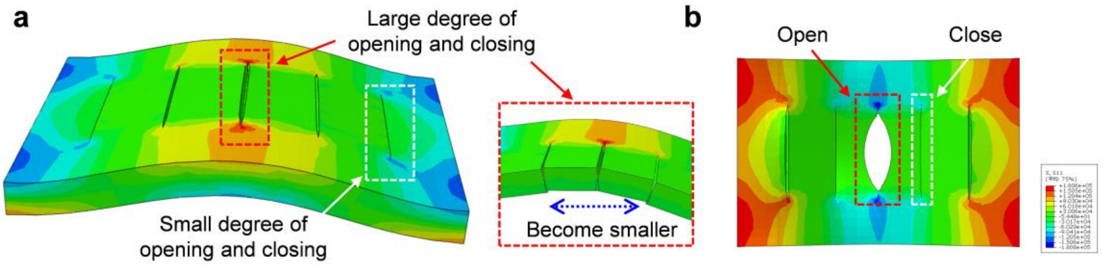

Fig. S11 Finite element analysis of the bionic flexible sensor. (**a**) Vibration stimulation of the sensor results in deformation of the parallel through-slit structure. Depending on the hysteresis of the material, different vibration frequencies correspond to different degrees of seam deformation. (**b**) Schematic diagram of Z-axis cross-section
